# Ninth International Medical Congress, Washington, D. C., September, 1887

**Published:** 1888-03

**Authors:** 


### NINTH INTERNATIONAL MEDICAL CONGRESS, WASHINGTON, D. C. SEPTEMBER, 1887.

### SECTION XVIII, DENTAL AND ORAL SURGERY.

REPORTED FOR THE INDEPENDENT PRACTITIONER, BY "MRS. M. W. J."

CONTINUED FROM PAGE 87.

### FRIDAY AFTERNOON SESSION.

Dr. W. W. Allport moved that, in order to hear all of the remaining papers, there be no more general discussion.

Dr. A. E. Baldwin hoped the motion would not prevail, as it would cut off those who might have valuable thoughts to offer. He thought it better to get all the good possible out of one paper than to hurry through many.

Dr. Atkinson agreed with Dr. Baldwin. There was no value in accumulating a great mass of material.

Dr. Allport withdrew his resolution.

A paper was then presented from Dr. E. Andrieu, Paris, president of the Odontological Society of France, on "The Sixth Year Molar." An abridged translation of the paper was read by Dr. L. D. Shepard, of Boston, Mass.

In this paper the first molar was considered as a "tooth of transition," whose period of usefulness is limited to the time of shedding the deciduous teeth. That it is not designed as a permanent tooth he considers proved by the fact that it originates in the epithelial layer like the other deciduous teeth, and not from a bud connected with the preceding teeth, the permanent second molar springing from the "deciduous" sixth year molar. The function of the latter he conceives to be limited to the work of defining the space to be occupied by the "teeth of replacement," and of maintaining the articulation at the desired height until the anterior teeth and the second molars are in place. After that he thinks it is not needed, and its retention injurious; that it should he extracted without hesitation, seventy-five out of every hundred decaying early and injuring the neighboring teeth as well as the general health. From his examination of one hundred mouths, he found only two adults with the four first molars in good condition; eleven with three; thirty-one with only one good one, and fifteen with some remains of first molars; in forty-one they were entirely gone. That this should be the case he thinks only to be expected from their inferior density, from the external configuration of the crown, from the acid fluid in which they are bathed during the period of displacement, and from the proximity of the second temporary molars "which are always decayed before they are shed." Their coefficient of resistance is less than in the other teeth whose density is greater. The acid condition of the mouth he attributes to the fact that "the interstices of the temporary teeth are packed with decaying, fermenting food," constituting them "sinks of infection."

The decay of the second temporary molar injures the first molar, which, in its turn, affects the permanent molar; its retention also crowds the space and leaves no room for the wisdom tooth; being crowded, it erupts with a facial inclination favoring the lodgment of food to the injury of the second molar. The removal of the sixth year molar banishes all other causes of deterioration. He concludes that the sixth year molar should be retained only when it is of superior structure, showing no signs of decay, and the jaws amply large to accommodate the thirty-two teeth without crowding, but these cases he finds very rare.

He finds, in most instances, abscesses from the decay of the first molar in the first year after eruption. When room is needed for regulating, the sixth year molar can always be spared. If the permanent central incisor erupts in front of or behind the arch, he would sacrifice the temporary lateral. Then, if the permanent lateral has not space, he recommends the sacrifice of the temporary canine. The sixth year molar having been extracted, when the temporary molars are shed or extracted the bicuspids will have plenty of room. "This rule makes success easy." The proper time to extract the sixth year molar he considers to be when the second molar is about to come through. If necessary, it should be filled and cared for until then. The recent fashion of no extractions has resulted in causing havoc in many mouths, with abscesses, swelled faces, and heads tied up, and dictates a return to the plan of extracting the sixth year molar.

The discussion of this paper was opened by Dr. L. D. Shepard. He said, had this paper not come from across the water, but been presented by one of our own people, it would not have been deemed fit to go on record as an exemplification of modern dentistry. He was sorry to have to pronounce such a severe judgment, but the paper advocated a return to the practice of twenty-five, or rather fifty years ago, when the sixth year molar was extracted promiscuously. The results of modern practice prove that with thirty-two teeth in the mouth, only due respect is paid to each one. No tooth, any more than any other suspected violator of the law, should be condemned without trial by jury; it should not be anathematized unless proved a culprit; the thirty-two teeth are all entitled to equal respect. Another fact to be recognized is that extraction is mutilation. Amputation is also mutilation, but when amputation is decided upon it is for a determined reason, and not because it is the right ear or the left, the right eye or the left, and not because it is the sixth year molar or the bicuspid, but the decision should be had upon the study of the laws governing the case. The extraction of a tooth may be a question of expediency, but it should depend upon a lesion of the tooth, the occlusion of the jaws, or the arrangement of the teeth. It should be as the result of some abnormal condition, not physiological, but pathological. Even admitting the sixth year molar to be a tooth of transition, a method of practice based upon one condition only has a very contracted foundation. If it is a tooth analogous to the temporary teeth, if a mere tooth of transition, why was no provision made for its self-extraction, by exfoliation or the absorption of its roots? This is sufficient argument to prove it a permanent tooth.

Dr. Andrieu's tables of the frequency of decay are analogous to tables of the deaths from small-pox if all smitten with the disease were allowed to die without any care, nursing or medicine. To judge fairly we should have the ratio of their preservation in adult life, when all preventive and remedial means have been employed. At the age of fifty years we will find more six year molars than bicuspids, where all have had the same care. It has as good a prospect as any other tooth, excepting only the canine. Hence, in his practice, Dr. Shepard said that he always insisted on giving the sixth year molar the same respectful consideration that he gave any other tooth, beginning with its eruption.

Dr. Paul DuBois, of the Dental School of Paris, said that he did not share Dr. Andrieu's opinions. He could not believe that the sixth year molar had the characteristics of a first tooth; it was the most feeble tooth, he thought, and should receive the earliest attention, and be extracted only at the last extremity. Its extraction changes the whole articulation. In the civilized races we find the jaws narrowed, with no place for the wisdom tooth; we should not aid that tendency to narrow the jaws still more, interfering with mastication.

Dr. Horton, of Cleveland, Ohio, instanced the cases of his two sons. For one he filled the sixth year molars as soon as they began to decay, and at the age of thirty-five he has his thirty-two teeth; for the other he extracted the sixth year molars at the age of eight or nine. But when the third molars erupted they found no room, but were forced into the masseter muscles, occasioning severe pain, and, at the age of thirty-four, as handsome third molars as were ever seen had to be extracted. Age, sex, the general condition of the mouth, the size of the jaws, must all be taken into consideration before extracting. Nature has given thirty-two teeth because she wants them all The temporary teeth should be filled with the first appearance of decay; before the age of three years is not uncommon now, where we have control of whole families.

Dr. Frank Abbott, of New York, said that in reference to the question whether the sixth year molar was a permanent or a temporary tooth, two points were essential. The assertion that they are not as well calcified as the second or third molars was entirely gratuitous. Under the microscope the temporary and permanent teeth are quite different. In the former the canaliculi are larger, but there is no such difference between the sixth year molar and the other permanent teeth. Dr. Andrieu assumes that it is a temporary tooth, or tooth of transition, and should be removed because it originates from an independent bud in the same manner as the first teeth. But there is this important difference—from the temporary teeth the bud goes down and the second tooth comes from underneath, but from the sixth year molar the bud projects horizontally for the second, and from the second to the third in the same way. They are all intended for permanent teeth, from the first to the last of the three.

A paper was read from Dr. Thomas David, Director of the Paris Dental School, on " Aphthous Stomatitis and its Origin."

Dr. DuBois then tendered an invitation to the members of the Section to attend the National Congress of Dentists in Paris, in 1889, which would be a grand occasion, where they would be received with a welcome such as he and his confrères had received at the present Congress. The profession in France were all united in tendering the invitation, and desirous of a large attendance.

Dr. John S. Marshall, of Chicago, read a paper entitled "Operation for the Cure of a Persistent Neuralgia of both Temporo-Maxillary Articulations, and Reflected Pain in the Right Brachial Plexus, of Eight Years' Standing, with Results." After enumerating a long list of causes of neuralgia, the case in hand was found to be a peculiar one, and due to mal-position of the parts from the removal of a large section of the maxillary bone, for an osteo-sarcoma, three years previously. The maxillary bone having been removed, from the first bicuspid nearly to the angle of the ascending ramus, an ugly cicatrix had been left, and the jaw carried back to the right until the median line was an inch from the centre. There had been fibrous union, but this afforded little support. The mouth could be opened only three-fourths of an inch, and the patient—a seamstress, forty-two years of age—suffered severe paroxysms of pain in the side of the jaw, extending to the shoulder and down the arm. She was wearing a full upper denture. The case was diagnosed by Dr. Marshall as one of phagedenic pericementitis, due to the contraction of cicatricial tissue, and he decided to make an operation for the relief of the mal-position of the jaw. Removing the fibrous tissue in the mouth, the jaw was carried back into proper position and the wound packed with sterilized sponge. The $\frac{1}{1000}$ bichloride of mercury solution was used as a mouth-wash every two hours, and it was hoped that the sponge would organize. As long as the sponge remained in place there was no recurrence of the pain, but suppuration took place and the sponge had to be removed, when the pain returned in her shoulder, but not in her arm. Another method was then tried. A gold crown was fitted to the bicuspid, to which one end of a gold rod was soldered, the other end having a screw thread fitting a hole drilled in the ramus. The rod was one-eighth inch in diameter and one and one-eighth inches long, and held the jaw in its normal position. An artificial bridge denture was then constructed to fill the space and counteract the pressure of the bicuspid holding the rod. The first operation was made in March. On the 17th of May an incision was made externally, in the line of the original cicatrix, with two objects in view; first, to try bone-grafting from the outside, and second, to get rid of the old scar tissue. A flap of periosteum was raised from the bone on one side, and from the ramus on the other, and sterilized sponge inserted, with drainage by strands of floss silk. This again suppurated, and the sponge was removed on the 29th. By the 17th of June the wound had healed, the screw in the ramus occasioning no inconvenience or irritation. On the 3d of September, however, in sneezing, the screw was displaced. The jaw had been carried back half an inch further, and the mouth opened much wider. The rod was consequently too short, and a half inch length of tube was soldered to the tooth, with a set-screw on the rod to hold it to the right length. In January the screw was still firm, with no inconvenience, but a marked improvement in the position and movements of the jaw, the mouth opening an inch and a half. An attempt at bone-grafting was again made, from the femur of a young rabbit. Twelve small pieces of bone, from two to six lines in length, were inserted in two rows across the gap. Every piece attached, and the wound healed without suppuration, and with no drainage; union remained perfect with the ramus, but at the other end necrosis set in after sixteen days, and further attempts were abandoned, owing to the debilitated condition of the patient. In July the screw was displaced in yawning, and the crowned bicuspid had become so loose it was removed. A heavy plate was then made of Weston's metal. There has been no recurrence of pain since May, 1886, but Dr. Marshall said he would have been better satisfied if the reproduction of bone had proved a success. The first sponge graft failed because of septic influences, it having been impossible to exclude the fluids of the mouth. The second failed from using pieces of bone larger than the tissues could nourish. This was an unfavorable opportunity for brilliant success, the patient being forty-two years old, and her vital energies much depressed from long suffering.

There are three principles to be borne in mind in bone grafting. First, thorough cleanliness, during and after the operation; second, pieces of bone must be very small and covered with periosteum; third, they must be taken from a young and growing subject. If he could have obtained a fœtus, or young child just dead, he would have tried human bone. The rabbit died under the effects of the anæsthetics used. The bone to be used must be immersed in bichloride solution $\frac{1}{500}$ at the temperature of the body for five minutes.

Dr. Atkinson expressed his acknowledgments to the author of the paper for his faithful delineation of details. He had laid down clearly the principles involved in bone or sponge grafting, and had exposed the folly of the old methods of using any kind of drainage. Whenever we use drainage we open the way for retrograde metamorphosis—material to be wept out. He regretted that there was not a whole day in which to discuss so valuable a contribution. There was a little of the holding to effete traditions and inane quotations in trying to define what neuralgia is. It is not neuralgia when it is due to compression. After some further discussion of this subject the following papers were read by title :

"Articulation of Artificial Teeth," by Dr. H. L. Cruttenden, of Northfield, Minn.

"Power in Dentistry," by Dr. W. St. Geo. Elliott, of London, England.

"Porcelain Crowns," by Dr. E. C. Moore, of Detroit, Mich.

Adjourned.

